# Exposure to Smoke During Development: Fetal Programming of Adult Disease

**DOI:** 10.1186/1617-9625-3-2-5

**Published:** 2006-08-15

**Authors:** Hugo T Bergen

**Affiliations:** 1 Dept. of Human Anatomy & Cell Science, University of Manitoba

## Abstract

It is well established that smoking has potent effects on a number of parameters including food intake, body weight, metabolism, and blood pressure. For example, it is well documented that 1) there is an inverse relationship between smoking and body weight, and 2) smoking cessation is associated with weight gain. However, there is increasing evidence that smoking can exert deleterious effects on energy balance through maternal exposure during fetal development. Specifically, there appears to be an increased incidence of metabolic disease (including obesity), and cardiovascular disease in children and adults that were exposed to smoke during fetal development. The present review will examine the relationship between maternal smoke and adult disease in offspring. The epidemiological studies highlighting this relationship will be reviewed as well as the experimental animal models that point to potential mechanisms underlying this relationship. A better understanding of how smoking effects changes in energy balance may lead to treatments to ameliorate the long-lasting effects of perinatal exposure to smoke as well as increasing the health benefits associated with smoking cessation.

## Smoking And The Fetal Origins Of Disease

### I. Introduction – Fetal Programming of Adult Disease

Recently there has been increased attention paid to the hypothesis that some diseases that have been considered to be diseases of adulthood (e.g., obesity, type II diabetes, cardiovascular disease, hypertension, and some cancers) may have their origins in fetal development. This is most commonly referred to as either the "developmental origins of disease" or the "fetal origins of disease" [1-7]. The hypothesis states that the susceptibility to develop some diseases is determined in part by the intrauterine and early postnatal environment, i.e., perturbations to the early environment (e.g., nutritional deficits) can significantly increase susceptibility to develop disease later in life. The association between poor fetal and newborn growth and the subsequent development of obesity is a commonly cited example of this phenomenon [7,8]. The epidemiological studies that reported this association led to the formulation of the "thrifty phenotype hypothesis" [9]. This hypothesis stated that a poor nutritional environment for the fetus, brought on by either malnutrition or placental dysfunction, can induce an adaptive response that will optimize growth and development later in life. The adaptive response (which may include changes in circulating hormones, receptor sensitivity, regulatory enzymes, central nervous system changes), would lead to potential increased survival of the adult individual under conditions of marginal nutritional supply. However, under conditions of nutritional abundance the result will be maladaptive with increased incidence of obesity, hyperlipidemia, hypertension and type II diabetes in adulthood.

One of the first and best documented examples of this phenomenon was the development of obesity in individuals 20 years after they were born during the Dutch famine of 1944–1945. Individuals were at greater risk of developing obesity following exposure to under-nutrition during late gestation and early post-natal periods [10]. The last 30 years has seen numerous epidemiological studies outlining a relationship between gestational nutrition together with birth weight (as a marker of impaired fetal growth) and subsequent diseases such as obesity, type II diabetes, hypertension and cardiovascular disease to name a few [1,5,11-15] Epidemiological studies have now been bolstered by an increasing number of experimental studies demonstrating a relationship between perinatal nutrition and/or birth weight, and adult disease [2,7,16-19]. The mechanism(s) responsible for translating fetal effects into adult disease are not well understood but several candidates include imprinting through epigenetic programming.

Most of the research, with respect to early programming of adult disease, has focused on nutritional challenges (under- and over-nutrition) on the subsequent development of adult diseases. However, there is increasing evidence that long-lived effects of perinatal perturbations may not be limited to nutritional influences but may also include other influences such as maternal smoking. The present review will focus on the effects of maternal smoking on the subsequent development of diseases in the offspring. It should be noted that cigarette smoke contains numerous components that are biologically active. Although nicotine is a dominant factor in this regard, other components of smoke may also be involved in mediating the detrimental effects of smoke on fetal development and the subsequent promotion of disease into adulthood.

### II. Smoking and Obesity

One of the first epidemiological studies that examined the effect of maternal smoking on the subsequent development of obesity was a birth cohort study of over 17,000 births and these individuals were tracked at 16 and 33 years of age [20]. This study identified a significant effect of maternal smoking on the subsequent development of non-diabetic obesity, as well as an effect on diabetes (see below). In the offspring of mothers that smoked during pregnancy, there was significant increase in the incidence of obesity (at 33 years of age) and the magnitude of the effect was greater in the offspring of heavy smokers than the offspring of medium smokers. Similar results were obtained in a study of over 6,000 children [21]. Specifically, there was a dose dependent association between maternal smoking during pregnancy and childhood obesity (5–7 yrs. of age) that was independent of a variety of lifestyle confounders or other risk factors for obesity. However, it is also known that maternal smoking can significantly impair fetal growth [22,23] and that low birth weight is itself associated with increased incidence of obesity later in life [24]. In this clinical scenario, i.e., obesity in individuals born small for gestational age, the post-natal and early developmental period is characterized by a phenomenon referred to as "catch-up growth"[22,24-26]. Therefore, obesity due to maternal smoking may be secondary to the effects of maternal smoking on fetal growth; i.e., maternal smoking produces smaller babies and smaller babies are more susceptible to develop a variety of adult diseases. However, it should be noted that the relationship between maternal smoking during pregnancy and overweight in children is a complex one. Specifically, a more recent epidemiological study reported that the likelihood of being overweight at 4.5 years was almost doubled as a result of maternal smoking during pregnancy. Interestingly, the effect of maternal smoking on overweight was seen in normal birth weight children that had the greatest weight gain in the first few months of life as well as in high birth weight children that had the least amount of weight gain after birth [27]. This suggests that maternal smoking may increase susceptibility to develop overweight and this can occur in normal birth weight as well as high birth weight children.

However, although catch-up growth may be a factor in the subsequent development of obesity, studies also suggest that the effect of maternal smoking on obesity in offspring can occur independently of catch-up growth [21]. It was reported that infants exposed to maternal smoke had lighter birth weights but as they grew into adolescence they tended to have greater body mass index and this tendency increased with age [28]. Importantly, studies have reported that the association between maternal smoking on obesity remains even when adjusted for birth weight; i.e, maternal smoking exerts an influence independent of its effects on fetal growth [21,28,29]. In a subsequent study it was also reported that the effects of maternal smoke on development of obesity was observed in children 5–7 years of age and that if mothers that otherwise smoked, abstained during pregnancy and then resumed smoking after childbirth, the association was no longer observed [30]. This clearly suggests that intrauterine exposure to maternal smoke, rather than other family or lifestyle factors, can exert profound influences on regulation of energy balance that can be detected as early as 3 to 5 years of age [30,31]. For example, the size of the effect of maternal smoking on childhood obesity was as great as that observed in studies linking obesity with frequent television viewing and playing video games [21].

In a study that examined the relationship of maternal smoking to obesity in American Indian children there was a significant effect of maternal smoking during pregnancy on overweight at three years of age [31]. It is also of interest to note that the effect was independent of birth weight (and mother's pre-pregnancy weight). Given that smoking has also been linked to low birth weight and that low birth weight has been linked to obesity; the dissociation between these two variables in this study indicates that the effect of smoking is not simply through an effect on birth weight [32,33].

An important issue regarding the effect of maternal smoke on fetal development and subsequent health effects is determining the sensitivity of the fetus to the detrimental effects of smoke. From a public health perspective, it is vital to know whether smoking is harmful to the fetus in the first trimester when women who smoke and do not know that they are pregnant, will be unintentionally exposing their fetuses to the harmful effects of smoke. In a recent study it was reported that there was no difference in the effect of maternal smoking between mothers that smoked throughout pregnancy and those that smoked only during the first trimester on subsequent development of obesity [34]. These results could not be explained by other potentially confounding factors such as breastfeeding, watching television, playing video games, and parental obesity. This suggests that during the first three months of pregnancy the fetus is particularly sensitive to the detrimental effects of exposure to maternal smoke. In a recent prospective study, maternal smoking during early pregnancy was associated with overweight at three years of age [35]. Furthermore, the effect was observed even when adjusted for other factors such as ethnicity, income, education and childhood diet. In contrast, an increased incidence of obesity was not observed in children of mothers who quit smoking prior to conception (as compared to never smokers). These studies suggest that the detrimental effects of maternal smoking on fetal development may occur in the first trimester. Therefore, it seems prudent that women should be encouraged to quit smoking before conception; as is the case with alcohol consumption.

### III. Smoking and Diabetes

One of the first epidemiological studies that identified a link between the effect of maternal smoking and the subsequent development of diabetes was a birth cohort study of over 17,000 births. In addition to identifying maternal smoking as a risk factor for obesity, this study identified a significant effect of maternal smoking on the subsequent development of early adult onset diabetes [20]. For example, if a mother smoked more than 10 cigarettes a day while pregnant, there was a four-fold greater chance that the offspring would develop diabetes, as compared to offspring that were not exposed to smoke. Moreover in a more recent study aimed at examining the effects of smoking in association with other drugs (alcohol and illicit drugs), prenatal exposure to nicotine, but not alcohol, had a significant effect on subsequent BMI and similar to other studies, a dose response relationship was detected [36]. Taken together, these epidemiological studies suggest that prenatal exposure to smoke has a long-lasting effect on body weight. Specifically, there is a greater incidence of overweight and obesity in those exposed to maternal smoke and that there is a significant dose-response relationship. The mechanism for the effect is not known however the authors speculated that it may be related to either fetal malnutrition or the toxicity of smoke on the fetus.

### IV. Smoking and Hypertension

In addition to nicotine's effect on the subsequent development of obesity, maternal smoking also appears to have an effect on the subsequent development of cardiovascular disease; specifically hypertension. A number of studies have provided evidence that low birth weight is associated with hypertension later in life [37]. Therefore, since smoking during pregnancy acts to decrease birth weight, the effects of smoking on subsequent hypertension could occur indirectly through effects on birth weight. In a prospective cohort study with over 1700 pregnant women, a significant effect of maternal smoking during pregnancy on blood pressure at six years of age was found and interestingly, this was not entirely due to the effect of smoking on birth weight [38]. In a second prospective study of over 3,800 children, a relationship between smoking during pregnancy and increased blood pressure at 5 years of age was reported and the effect was independent of birth weight [39].

While the above studies highlight the detrimental effects of maternal smoking on the subsequent susceptibility to develop obesity, diabetes, and hypertension, the mechanism underlying this effect is not well understood and a number of possibilities have been proposed. Clearly, a better understanding of how maternal smoking, and/or nicotine, effects changes in energy balance, metabolism, and blood pressure, may lead to treatments to ameliorate the long-lasting effects of perinatal exposure to smoke as well as increasing the health benefits associated with smoking cessation. In any case, recommendations to stop smoking are particularly relevant in women that are planning or attempting to become pregnant.

### V. Smoking and Fetal Origins of Disease: Potential Mechanisms

#### A. Decreased fetal growth

As mentioned above, a number of studies have found evidence of a link between low birth weight and subsequent development of obesity (Reviewed in [2,7,40,41]). Since maternal smoking is associated with decreased fetal growth, the increased incidence of obesity may be a secondary result of being born small for gestational age. However it should be noted that while low birth weight may play a role in subsequent disease processes, there is evidence that the detrimental effects of smoking may occur independently of low birth weight (see above). In any case, the mechanism(s) underlying the effect of nicotine on birth weight is (are) not well understood and a number of possibilities clearly exist. The possibilities highlighted below are neither exhaustive nor mutually exclusive.

##### 1. Low birth weight is due to smoking-derived nicotine effects on maternal appetite and energy expenditure

Nicotine is considered to have an inhibitory effect on body weight gain. This is supported by the association that smokers weigh less than non-smokers and cessation of smoking is accompanied by significant weight gain [42]. There is evidence to suggest that the mechanism for nicotine's effect on body weight may involve both an increase in energy expenditure and a decrease in food intake [43-48]. Therefore, during pregnancy the combination of nicotine-induced suppression of maternal food intake and increased energy expenditure may collaborate to be sufficient to produce poor or under nutrition which then leads to decreased fetal growth. The effect of smoking appears to be an effect of intra-uterine growth retardation and not an effect on pre-term delivery [32].

##### 2. Low birth weight is due to detrimental effects of nicotine on placental structure and function

A second possible mechanism responsible for smoking's effect on decreased fetal growth may be at the level of the placenta; i.e., through impaired delivery of oxygen and/or nutrients to the fetus. Nicotine via activation of nicotinic acetylcholine receptors, can exert a vasoconstrictive effect on placental arterial supply leading to attenuation of oxygen delivery to the fetus which in turn could lead to decreased fetal growth [49-51]. Smoking is also associated with increased carbon monoxide in maternal blood which in turn reduces oxygen delivery to the fetus leading to decreased fetal growth. Smoking decreases uterine blood flow to the placenta which could play a role in decreased fetal growth observed produced in the smoking pregnant woman [50,52,53]. There are also a number of studies that have reported that smoking is associated with marked changes in placental structure and function. For example, smoking is associated with a significant increase in the thickness of the villous membrane which could predictably lead to a decrease in gas and nutrient exchange across the placenta and impaired fetal growth [54,55]. Smoking also exerts detrimental effects on the trophoblast component of the placenta as well as decreasing the area for diffusion between the maternal blood and fetal blood [56-59]. For example, trophoblast cell differentiation is impaired with maternal smoking and this effect can occur early in the development of the placenta [58,59]. Structural changes of the placenta may act to decrease gas exchange, as well as nutrient exchange (e.g., amino acid transport) across the placenta [60] Taken together it is clear that smoking during pregnancy has significant effects on placental development that leads to significant changes in structure and function that may result in detrimental effects on fetal growth and well-being

##### 3. Low birth weight is due to detrimental effects of nicotine on fetal metabolism

A third possibility is that nicotine or other constituents of smoke may have detrimental or toxic effects on fetal metabolism that impair growth of the fetus. Nicotine readily crosses the placenta and therefore it could potentially exert direct effects on fetal tissues [49]. For example, it is becoming increasingly evident that the endocrine status of the fetus is altered significantly in response to maternal smoking [61,62]. In particular, leptin, growth hormone and insulin-like growth factor (IGF) levels in the fetal compartment are altered in response to maternal smoke [61]. It was suggested that decreased IGF levels detected in cord blood associated with maternal smoking may play a role in limiting fetal growth [62]. Of particular interest is the effect of maternal smoke and/or nicotine on leptin levels present in cord blood. As discussed below, leptin may exert profound influences on development of the neural systems underlying regulation of appetite and metabolism. Taken together, it is clear that maternal smoking may exert potent and detrimental effects on fetal growth via a number of mechanisms.

#### B. Perturbations in Central Regulatory Circuits

Most of the studies exploring potential mechanisms underlying smoking and disease later in life have focused on obesity. It is well established from animal studies that nicotine can act within the central nervous system to decrease food intake and body weight [63-66]. Although most studies examining nicotine's effect on energy balance are acute studies performed in adult animals, there is an increasing evidence that exposure to nicotine during development can produce long lasting perturbations in brain neurochemistry including dopamine, serotonin, acetylcholine, and a variety of neuropeptides, as well as leptin receptors [67-77]. These changes could potentially lead to long term changes in the neural regulation of energy balance, i.e., nicotine either directly or indirectly may alter appetite or self regulation of food intake in infants exposed to maternal smoke. It has been proposed that smoking acts by means of metabolic imprinting on the system controlling food intake and satiety. A number of rodent studies have provided evidence in support of the hypothesis that maternal nicotine has long-lasting effects on neurotransmitter systems that are implicated in the regulation of energy balance. In addition there is recent evidence suggesting that programming of the neural circuits that regulate energy balance occurs in the perinatal period and that metabolic or nutritional deficits result in altered regulation of energy balance, including appetite [78,79]. For example, a study in Rhesus monkeys demonstrated that maternal nicotine exposure had a significant effect on gene expression of neuropeptides (neuropeptide Y and proopiomelanocortin) in the hypothalamus [74]. It is well established that these hypothalamic neuropeptides can exert potent effects on the regulation of energy balance and it was proposed that altered regulation of these neuropeptides may play a role in decreased body weight associated with maternal smoking. Interestingly, it was also reported that circulating leptin levels were reduced following nicotine treatment, similar to the situation that occurs in humans [61,74]. Decreased leptin levels in response to nicotine is of particular interest since a number of recent studies have suggested that leptin may play a critical role in the development of brain circuits that regulate energy balance [80-83]. In genetically obese mice that lack leptin (i.e., ob/ob mice), exogenous administration of leptin had marked effects on synaptic contacts between excitatory and inhibitory hypothalamic neurons that regulate energy balance [82]. The importance of leptin in development is further bolstered by recent studies demonstrating that treatment with leptin during the perinatal period can offset developmental programming that occurs as a result of maternal undernourishment [84]. Clearly, the role of leptin as a hormone that regulates neuronal development is a topic of increasing interest and if nicotine alters circulating leptin during development, this could have long-term consequences [85]. In this regard it is also interesting to note that under-nutrition of mouse dams (which decreases circulating leptin) was associated with obesity in the pups when they were subsequently fed a high-fat diet [86]. This may be a mechanism by which nutritional perturbations in the perinatal period, for example brought on by maternal smoking, may have long-lasting effects. Taken together these results suggest that smoking may have long lasting effects on the neural circuits responsible for regulating food intake and metabolism.

### VI. Conclusion

It is becoming increasingly clear that fetal exposure to nicotine has numerous consequences to the detriment of the health of the fetus and that these effects may last well into adulthood. These effects include increased incidence of obesity, cardiovascular disease, and non-insulin dependent diabetes mellitus. It is not well understood how smoking may produce these long-lasting effects but it is evident that the fetal environment is of tremendous importance during the developmental period in determining health throughout the life of the individual. In any case, women who are planning a pregnancy now have additional reasons to stop smoking in addition to the health benefits that they will receive from nicotine abstinence.

## Competing interests

The authors declare that they have no competing interests.
